# BRAF-targeted therapy in non-melanomatous BRAF-mutant tumours: a systematic review of broad but histology-modulated efficacy

**DOI:** 10.1007/s10147-026-03079-y

**Published:** 2026-06-11

**Authors:** Yael R Lefkovits, Luke S McLean, Mark R Middleton, David M Thomas

**Affiliations:** 1 Department of Medical Oncology, St Vincent’s Hospital, Melbourne, Australia; 2 Department of Oncology, Oxford University, Oxford, UK; 3 Department of Medical Oncology, Peter MacCallum Cancer Centre, Melbourne, Australia; 4 Department of Medicine, University of Melbourne, Melbourne, Australia; 5 Centre for Molecular Oncology, School of Biomedical Sciences, University of New South Wales, Sydney, Australia

**Keywords:** BRAF, Tumour-agnostic, Precision oncology

## Abstract

**Background:**

Tumour agnostic therapies represent a paradigm shift in oncology. BRAF inhibitors have demonstrated efficacy in melanoma. However, their role in non-melanomatous cancers was originally contentious. This systematic review aims to evaluate the safety and efficacy of BRAF-targeted therapies, both as monotherapy and in combination with other targeted therapies, in BRAF-mutated, non-melanomatous tumours.

**Methods:**

A systematic search of MEDLINE, PubMed, EMBASE and Cochrane CENTRAL was conducted from January 2010 to January 2025. Eligible studies included prospective and retrospective trials as well as cohort studies assessing BRAF inhibitors in non-melanomatous cancers. Primary outcomes included objective response rate, progression free survival, duration of response and overall survival.

**Results:**

Thirty-six studies comprising 3141 patients were included, spanning over 14 tumour types. The majority (78%) were phase II basket trials. Colorectal (n = 1569, 50%), lung (n = 756, 24%) and thyroid (n = 114, 4%) were the most commonly represented tumour types. Combination therapy (e.g., BRAF and MEK inhibition concurrently) demonstrated superior efficacy to monotherapy (e.g. ORR 45–51% v 5–20% in colorectal cancer), with efficacy varying by histology. Toxicity profiles were consistent with known class effects and higher with more frequent ≥ grade 3 adverse events in combination therapy compared with monotherapy. Most studies included exhibited a moderate risk of bias due to single-arm design and lack of randomisation.

**Conclusion:**

Although BRAF-targeted therapies have been positioned within the tumour-agnostic paradigm, the available evidence suggests clinically meaningful activity across multiple non-melanomatous BRAF-mutant tumours that is nevertheless modified by tumour lineage, biological context, and treatment strategy.

## Introduction

### Background

Effective, molecularly-guided treatments, targeting a common molecular aberration, represent a paradigm shift in oncology, theoretically rendering organ-based classifications of limited use and replacing it with a molecular based definition of tumours [1]. This paradigm shift has facilitated the transition to ‘targeted therapies’ whereby the treatments are selective for molecular alterations, rather than cancer histotypes [2].

This concept has particular value for patients with rare cancers and cancers of unknown primary site (CUP), who may stand to benefit significantly from tumour-agnostic approaches [3]. Both groups have been disenfranchised by a therapeutic strategy based on tissue of origin, and have high levels of unmet need. Unfortunately, survival for patients with metastatic CUP remains poor, at approximately 20% at 1 year, and this has not improved considerably over the past few decades [4, 5]. Similarly, rare cancers often have poor survival outcomes, with 5-year relative survival for all rare cancers together varying from 40 to 55% depending on age, location and case-mix [6–9]. Despite their individual rarity, when considered collectively, rare cancers constitute a substantial proportion of the global cancer burden and almost 25% of cancer cases globally [10, 11].

### Approval of tumour-agnostic therapies

The promise of tumour-agnostic treatment began with the US Food and Drug Administration’s (FDA) approval of pembrolizumab for mismatch repair deficient (MMRd) or microsatellite instability high (MSI-h) tumours that had progressed after prior lines of treatment [12, 13]. Since then, larotrectinib received conditional FDA approval for patients with tumours harbouring a neurotrophic tyrosine receptor kinase (NTRK) gene fusion [14]. Subsequent therapies such as entrectinib have expanded this category, emerging as the third, FDA approved, tumour-agnostic treatment [15]. Since then, five additional drugs have received accelerated FDA approval for tumour-agnostic indications [16–18].

### BRAF inhibition

BRAF mutations are present in approximately 5–15% of all cancers, most commonly at the V600 locus [19–21]. These include between 5 and 15% of colorectal cancers [22, 23]. 1–5% of NSCLC [24] and up to 50% of thyroid cancers [25]. Over time, BRAF inhibition has expanded to use in several cancers including NSCLC, high and low-grade gliomas and anaplastic thyroid cancer [26].

BRAF activation is a key component of the mitogen-activated protein kinase (MAPK) cellular signalling pathway, promoting cell proliferation and survival [27]. Mutations in these pathways can lead to unrestrained activation of the downstream kinases and unchecked cell proliferation can lead to the formation of oncogenic features in many tumours [28].

Vemurafenib was the first FDA approved BRAF inhibitor, licensed in 2011 as monotherapy for unresectable or metastatic melanoma harbouring the BRAF V600E mutation [29]. Similarly, dabrafenib was FDA approved as a monotherapy in 2013 for the same indication. More recently, tovorafenib, a novel, type II RAF inhibitor, has been approved by the FDA for use in relapsed or refractory paediatric low-grade glioma, based on the phase 2 FIREFLY-1 trial [30, 31].

However, neither encorafenib, vemurafenib or dabrafenib have FDA approval as single agents in non-melanomatous, cancers. The lack of single agent activity outside melanoma has been attributed to feedback activation of alternative pathways, such as EGFR signalling in colorectal cancer [32].

The introduction of combination therapy has vastly broadened the clinical utility of BRAF inhibition. The addition of trametinib (MEK inhibitor) to dabrafenib, cetuximab (an EGFR inhibitor) or binimetinib (MEK inhibitor) to encorafenib and cobimetinib (a MEK inhibitor) to vemurafenib, has helped overcome aforementioned resistance mechanisms and broadened the clinical utility across various tumours [26, 33–35].

The FDA granted accelerated approval of dabrafenib and trametinib combined as a tumour-agnostic treatment for metastatic tumours harbouring BRAF V600E mutations [21, 31]. However, dabrafenib only has conditional FDA approval in combination with trametinib and only in the setting of mutations in BRAF V600E. Furthermore, no other BRAF inhibitor has been approved for use in a tumour-agnostic setting.

### Tumour agnostic applications of BRAF inhibitors

Vemurafenib monotherapy was assessed in one of the first tumour-agnostic, histology-independent trials – the VE-BASKET study. This trial investigated BRAF inhibition in multiple tumour types including NSCLC, biliary tract cancers, Erdheim–Chester disease (ECD), anaplastic thyroid cancer, clear cell sarcoma and pleomorphic xanthoastrocytoma [36]. The initial results were impressive, demonstrating a response rate of 42% [95% confidence interval (CI): 20–67] in NSCLC and anecdotal responses in the smaller tumour groups including pleomorphic xanthoastrocytoma and anaplastic thyroid cancer [36]. These unprecedented results highlighted BRAF mutations as a possible tumour-agnostic target.

Several clinical trials including the VE-BASKET study, ROAR trial (Rare Oncology Agnostic Research) and the NCI-MATCH studies, continue to explore the tumour-agnostic efficacy of BRAF inhibitors [21, 36, 37]. Early data from these basket trials demonstrated consistent responses to BRAF inhibition across > 20 different BRAF V600-mutated tumour types [38].

While narrative reviews have been published on the tumour-agnostic efficacy and tumour agnostic activity of BRAF inhibitors, there are no published systematic reviews to date examining their use in non-melanomatous cancers and as a tumour-agnostic therapy, both as standalone medications, and in combination with other therapies such as MEK or EGFR inhibitors.

This systematic review therefore aims to evaluate the evidence supporting the safety and efficacy of BRAF inhibition in non-melanomatous cancers, with a particular focus on their potential as a tumour-agnostic therapy. By synthesising the data from diverse studies, this review seeks to guide the development of targeted therapeutic strategies for patients with BRAF-mutated cancers, regardless of the tissue of origin.

## Methods and materials

The systematic review was performed using methods of the Cochrane Database of Systematic reviews and reported in accordance with the Preferred Reporting Items for Systematic Review and Meta-Analysis (PRISMA) guidelines.

### Data sources and search strategy

The systematic review was conducted using text words and medical subject heading terms (MeSH) related to targeted therapy for BRAF-mutated cancers, excluding melanoma. PubMed, Cochrane Library and EMBASE via Ovid were searched in addition to the reference lists of relevant articles. Studies were searched from 3rd January 2025, and all articles from January 1st 2010, until search date, were included.

A clinical librarian developed the systematic review search strategy in consultation with senior authors. The titles and abstracts of all citations obtained through the search were independently reviewed for eligibility using Rayyan software. Full-text review was conducted by one author. The Cochrane Risk of Bias 2 tool was applied to randomised trials, while the ROBINS-I tool was used for non-randomized studies.

**Table:** 

Population	Patients diagnosed with non-melanomatous cancers harbouring a BRAF mutation
Intervention	BRAF inhibitor therapy (Vemurafenib, Dabrafenib, Encorafenib or Tovorafenib) as monotherapy or in combination with other therapies
Comparison	Variable: standard of care, no comparison
Outcome	Efficacy outcomes including progression free survival, overall survival, overall response rate and duration of response

### Inclusion/exclusion criteria

We included prospective trials, both randomised and non-randomised clinical trials, retrospective cohorts and case–control studies. Case series, case reviews and trials with fewer than 5 participants were not included, to align with the ESMO ETAC-S guidelines. We excluded review articles, animal studies, editorials and in-vitro studies. Phase I trials were not included given this review focuses on treatment efficacy and comparative effectiveness. The subject was limited to humans and articles published in English.

We included studies examining patients with cancers harbouring any type of BRAF mutations, excluding melanoma alone.

### Eligibility criteria

Subgroup analyses from studies reporting multiple cancer types or targetable mutations (including melanoma, haematological malignancies, and BRAF-wild type cancers) were included if they involved distinct subgroups of > 5 patients with BRAF-mutated, non-melanomatous cancers. All patients were included regardless of sex or age. Eligible comparators included placebo, no treatment, standard of care, or chemo/immunotherapy regimens.

### Intervention

Studies were included if patients received BRAF inhibitors either as monotherapy or in combination with other chemoimmunotherapy agents. For the purposes of this review, BRAF inhibitors were defined as drugs that target the BRAF pathway and alter tumour TME. Approved BRAF inhibitors included vemurafenib, dabrafenib, encorafenib, and tovorafenib. While these agents are often combined with MEK inhibitors, selective MEK1/2 inhibitors (e.g., selumetinib, cobimetinib), without concurrent BRAF inhibition, were not included.

### Data extraction

Data were extracted in adherence to PRISMA guidelines and included study details (e.g., first author, publication date, study design, and trial phase), efficacy outcomes, and toxicity data. The extracted information was compiled in Microsoft Excel. Risk of bias was assessed using the Cochrane Risk of Bias 2 tool for randomized controlled trials and the ROBINS-I tool for non-randomized studies. While risk of bias tools was applied, formal scoring was limited due to the predominance of non-randomised, phase II trials.

### Data synthesis

Qualitative pooling was explored, but meta-analysis was not undertaken because of substantial clinical and methodological heterogeneity across studies including variation in histology, BRAF mutation subtype, treatment regimen, line of therapy, study design, sample size and reporting of efficacy endpoints. Under these conditions, pooled effect estimates were considered unlikely to be robustly clinically interpretable. Instead, we performed a structured qualitative synthesis stratified by tumour type and treatment approach.

## Results

### Study selection and characteristics

**Fig. 1:**
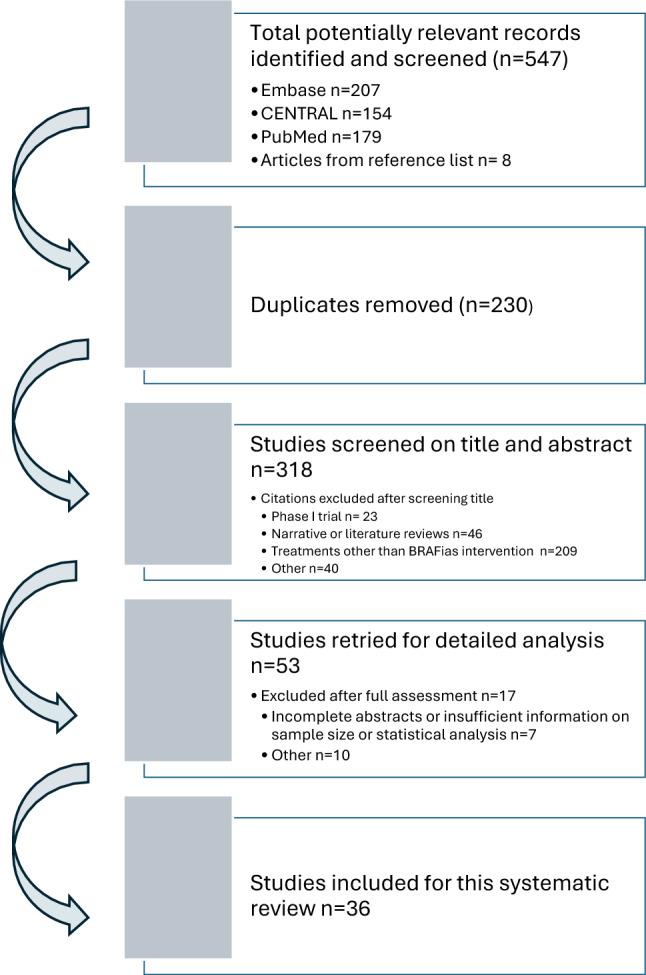
PRISMA flow diagram

A total of 547 articles were identified through database searching and manual review, with 230 duplicates removed, leaving 318 records screened on title and abstract. After excluding non-relevant studies, 53 full-text articles were reviewed in detail, with 17 excluded for reasons including small sample size or incomplete data Table 1. Ultimately, 36 studies were included in this systematic review (Fig. 1).

**Table 1:** Overall risk of bias profile of included studies

Domain	Evidence from this review	Likely implication
Randomisation and comparators	Only 3 of 36 included studies were randomised; most studies lacked comparator arms	Treatment effects may appear more favourable in single-arm settings because benefit cannot be directly compared with standard care or matched controls
Study phase and design	The evidence base was dominated by early-phase studies, particularly phase II trials, with additional retrospective and prospective cohort data	Efficacy signals are encouraging but are more vulnerable to bias and less definitive than evidence from larger randomised trials
Selection and confounding	Many included studies were non-randomised, and several efficacy estimates were drawn from basket cohorts or subgroup analyses	Selection bias and confounding may influence observed response rates, particularly in highly selected molecular subgroups
Precision	Rare histologies were frequently represented by very small patient numbers, and the review notes limited evaluable cases in several tumour types	Estimates in rare tumours are imprecise and should be interpreted as hypothesis-generating rather than definitive
Outcome reporting	ORR, PFS, OS and DOR were not uniformly reported across studies, and the manuscript acknowledges inconsistency in endpoint reporting	Direct cross-study comparison is limited, and heterogeneity in reporting reduces the strength of any overall efficacy synthesis
Overall assessment	Risk-of-bias tools were applied, but formal scoring was limited by the predominance of non-randomised phase II studies	Overall, the evidence base is best regarded as having moderate risk of bias, requiring cautious interpretation of efficacy estimates

The 36 identified trials were published between 2016 and 2024. As seen in Table 2, only three were randomised (8%). Phase I trials were excluded. There were only two -phase III trials which met inclusion criteria (5%). There were 28 phase II trials (78%), 3 retrospective cohort studies (8%) and 1 prospective cohort study (3%).

**Table 2:** Single tumour type studies (including single cohort analysis from larger basket trials)

Tumour group	Study	Phase	Random	Mutation
Colorectal	Koptez [39]	3	Y	V600E
	Koptez [40]	2	Y	V600E
	Cutsem [41]	2	N	V600E
	Koptez [42]	3	Y	V600E
	Ducreux [43]	2	N	Not specified
	Fernandez Montes [44]	Retrospective	N	V600E
	Klute [45]	2	N	V600E and K601E
	Chen [46]	Prospective	N	V600E
	Gallois [47]	Retrospective	N	V600E
	Tian [48]	2	N	V600E
CNS	Wen [49]	2	N	V600E
	Kaley [50]	2	N	V600
	Hargrave [51]	2	N	V600
	Hargrave [52]	2	N	V600
	Nobre [53]	Retrospective	N	V600E
	Kilburn [30]	2	N	Multiple
Lung cancer	Subbiah [54]	2	N	V600
	Riely [55]	2	N	V600E
	Planchard [56]	2	N	V600E
	Planchard [57]	2	N	V600E
	Planchard [58]	2	N	V600E
	Lin [59]	2	N	V600
	Swalduz [60]	2	N	V600E
	Auliac [61]	2	N	V600E
	Mazieres [62]	2	N	V600 and non V600
Thyroid	Subbiah [63]	2	N	V600E
	Tahara [64]	2	N	V600
	Brose [65]	2	N	V600E
Biliary Tract	Subbiah [66]	2	N	V600E
Craniopharyngioma	Brastiasnos [67]	2	N	Any BRAF
Basket (multiple tumour types)	Hyman [36]	2	N	V600E/G and unknown
	L Tan [68]	2	N	V600E
	Tahara [69]	2	N	V600E/R
	Blay [70]	2	N	V600 and non-V600
	Salama [37]	2	N	V600E
	Subbiah [71]	2	N	V600

### Risk of bias assessment

Risk of bias was assessed using RoB2 for randomised studies and ROBINS-I for non-randomised studies, as discussed in Table 1. Overall, the certainty of the evidence base was limited by the predominance of early-phase, non-randomised studies. Only 3 of the 36 included studies were randomised, while most were phase II trials, basket studies, or observational cohorts without comparator arms. These designs increase susceptibility to selection bias, imprecision, and overestimation of treatment effect, particularly in rare histologies and small subgroup analyses. Accordingly, the overall risk of bias across included studies was considered moderate, and efficacy outcomes should be interpreted cautiously in the context of limited randomised evidence and heterogeneous study designs.

### Patient and tumour characteristics

A total of 3141 patients were included across all trials. These patients spanned a diverse range of tumour types, as outlined below in Table 3. The largest tumour group was the gastrointestinal tract, comprising 1569 patients (50%) primarily with colorectal cancer. Lung cancers were the second most common, totalling 756 (24%) patients, of which the majority were NSCLC, although some small cell cases were included. Fewer than 1% of cases fell into the categories of anal carcinoma**,** ameloblastoma, or cancer of unknown primary**.**

**Table 3:** Breakdown of patients by tumour type across all studies

Tumour type	No (%)
*GI tract*	1569 (50%)
Colorectal	1569
*Thyroid*	114 (4%)
Anaplastic	40
Differentiated thyroid cancer	17
Papillary	51
Histology not specified	6
*Lung*	756 (24%)
NSCLC	751
Histology not specified	5
*Central nervous system*	416 (13%)
High grade glioma	98
Low grade glioma	238
Glioblastoma	16
Astrocytoma	24
Anaplastic ganglioma	3
Histology not specified	13
Glioma grade not specified	24
*Ovarian*	15 (< 1%)
Low grade serous ovarian cancer	2
Histology not specified	13
*Biliary tract*	73 (2%)
Histology not specified	43
Cholangiocarcinoma	30
*Craniopharyngioma*	16 (< 1%)
Papillary	16
Pancreatic	5 (< 1%)
Histology not specified	5
*Anal*	1 (< 1%)
Adenocarcinoma	1
*Neuroendocrine*	7 (< 1%)
GI origin	4
Location not specified	3
*Haematological*	86 (3%)
Histiocytosis	53
Multiple myeloma	5
Hairy cell leukemia	27
Histology not specified	1
*Ameloblastoma*	1 (< 1%)
Mandibular	1
Peritoneal	1 (< 1%)
Cancer unknown primary	5 (< 1%)
Sarcoma	9 (< 1%)
Other/otherwise not specified	68 (2%)

Most studies looked only at tumours with the BRAF V600E mutation, as shown in Table 2. One study didn’t specify the subtype of BRAF mutations identified and 7 studies included non-V600 mutations, although V600E mutations still made up the majority of included study participants in these trials.

### Therapeutic efficacy—monotherapy

Monotherapy for the treatment of BRAF mutated cancers demonstrated variable efficacy across tumour types, with an ORR ranging from 25 to 67%. Table 4 outlines treatment efficacy of monotherapy (most commonly vemurafenib) in single-arm, single tumour studies.

**Table 4:** End points (single-arm, single-tumour studies of BRAF monotherapy)

Study	BRAFi	ORR [95% CI]	PFS [95% CI]
*Central nervous system cancers*			
Kaley [50]	Vemurafenib	25% [10–47]	5.5 mo [3.7–9.6 mo]
Hargrave [52]	Dabrafenib	44% [26–62]	1 year 85% [64%–94%]
Nobre [53]	Dabrafenib or vemurafenib	PLGG 42% [NS] PHGG 35% [NS]	NA
Kilburn [30]	Tovorafenib	67% [54–78]	19.4 mo [16.9–NR]
*Lung cancer*			
Subbiah [54]	Vemurafenib	37.1% [25.2–50.3]	15.4 mo [9.6–22.8]
Planchard [57]	Dabrafenib	33% [ 23·1–44·9]	5.5 mo [3.4–7.3]
Mazieres [62]	Vemurafenib	44.9% [35.2–54.8]	5.2 mo [3.8–6.8]
*Thyroid cancer*			
Brose [65]	Vemurafenib	ORR NS Best OR 38.5% [20.2–59.4]	Cohort 1 (not pre-treated with VEGF therapy): 18.2mo [15.5–29.3] Cohort 2 (pre-treated with VEGF therapy): 8.9mo [5.5–NE]

PLGG = paediatric low-grade glioma; NR = not reached; NA = not available; NE = Not evaluable; mo = months; NS = not stated; PHGG = paediatric high-grade glioma

Among single-arm trials of BRAF monotherapy in CNS tumours, efficacy varied between trials and tumour subtypes, with tovorafenib demonstrating the highest ORR of 67% in paediatric low-grade gliomas [30]. In Nobre et al.’s study, although participants were treated with either dabrafenib or vemurafenib, there were no head-to-head comparisons made between the two agents [53]. Notably, after discontinuation of BRAF inhibition, 76.5% of patients with paediatric low-grade gliomas experienced rapid progression. However, upon rechallenge with BRAF inhibition, 90% achieved an objective response, suggesting that tumour control may be influenced by continuous BRAF inhibition [53].

The ORR with BRAF monotherapy in lung cancers was modest, with the choice of agent not appearing to greatly influence efficacy. Efficacy did appear to vary depending on pre-treatment status, with the median PFS in Subbiah’s study varying depending on whether participants had been previously treated or not (15.4 months [8.2–22.6] vs. NE respectively) [54]. Similarly, the efficacy of BRAF monotherapy in thyroid cancers was modest, with the best overall response in Brose et al.’s study being 38.5% [20.2–59.4] [65].

In comparison to the single-arm studies, basket trials demonstrated consistent responses to BRAF inhibition in BRAF mutated tumours, particularly among NSCLC and thyroid cancers (as outlined in Table 5). The VE-BASKET trial assessed vemurafenib across multiple tumour types including NSCLC, cholangiocarcinoma and anaplastic thyroid cancer [71]. ORR were consistent across certain tumour types including 42% in NSCLC, highlighting potential tumour-agnostic utility.

**Table 5:** Basket trials efficacy (monotherapy)

Study	BRAFi	Major tumour types	Outcome [95% CI]
Blay [70]	Vemurafenib 960 mg BD	Primary tumour, N (%), n = 97 Hairy cell leukemia 27 (28) Glioblastoma 10 (10) Cholangiocarcinoma 9 (10) ECD and histiocytosis 8 (8) Ovarian 6 (6) Thyroid 6 (6) Xanthoastrocytoma 5 (5) Ganglioma 4 (4) Sarcoma 3 (3) Bladder 2 (2) Multiple myeloma 2 (2) Nephroblastoma 2 (2) Gastrointestinal adenocarcinoma 1 (1) Lymphoid hemopathy 2 (2) Prostate 2 (2) Melanoma 5 (5) Others* 3 (3)	ORR: 52.0% [42.2–61.8] mPFS 8.8 mo [7.8–13.1] DCR n/N (%) = 78/97 (80.2)
Subbiah [71]	Vemurafenib 960 mg BD	Primary tumour, N (%), n = 172 NSCLC 63 (37) Histiocytosis 27 (17) Glioma 24 (14) Anaplastic thyroid 12 (7) Colorectal cancer 10 (6) Cholangiocarcinoma 9 (5) Sarcoma 6 (3) CUP 5 (3) Ovarian 4 (2) Neuroendocrine, NOS 3 (2) Pancreatic 3 (2) Others 6 (3) Others include neuroendocrine, head and neck, cervix, squamous cell and oesophageal	ORR: 32.6% [25.6–40.1] DOR: 13.1 mo [8–22.1] PFS: 5.8 mo [5.4–7.6] OS: 17.6 mo [13–28.2]

Values are rounded to the nearest 0.1 decimal point. ORR = overall response rate; OS = overall survival; DOR = duration of response; PFS = progression free survival; OD = once daily; BD = twice daily; DCR = disease control rate; PD = progressive disease; SD = stable disease; CR = complete response; PR = partial response; NOS = not otherwise specified. *Other includes neuroendocrine, head & neck, cervix, squamous and oesophageal

The VE-BASKET trial was among the earliest studies to apply histology-independent principles and importantly highlighted the potential for durable responses in select tumour types, supporting the feasibility of a tumour-agnostic approach [71]. However, there were some histological subtypes which demonstrated more resistance to monotherapy. For example, patients with colon cancer were excluded from the outset following an interim analysis in 11 patients demonstrating insufficient activity [71]. This suggests that tumour biology and co-mutational pathways may play a role in the efficacy of monotherapy.

In Blay et al.’s study assessing efficacy of BRAF inhibition in cancers other than melanoma and NSCLC, the long-term efficacy of single-agent vemurafenib was identified [70]. Although durable responses were seen across the global cohort, specific histological subtypes demonstrated particularly efficacious outcomes including xanthoastrocytoma (ORR 50%), ovarian cancer (ORR 50%) and sarcoma (ORR 60%). However, the sample sizes in these cohorts were small [70, 72].

### Therapeutic efficacy-combination therapy

Across several tumour types, combination therapies generally had greater response rates compared to monotherapy in single-arm, single-tumour studies (as demonstrated in Table 6). In colorectal cancer treated with doublet or triplet therapy, ORRs ranged from 20% to 50% with a median PFS spanning 15.7 weeks–5.8 months. Cutsem et al. reported an ORR of 47.4% with triplet therapy [41].

**Table 6:** End points (single-arm, single-tumour studies of BRAF therapy in combination with other therapies)

Study	BRAFi	Additional regimen	ORR [95% CI]	PFS [95% CI]
*Colorectal cancer*				
Cutsem [41]	Encorafenib	Binimetinib + cetuximab	47.4% [37–57.9]	5.8 mo [4.6–6.6]
Ducreux [43]	Vemurafenib	5FU/LV + cetuximab	20–50% [NS]	NA
Fernandez Montes [44]	Encorafenib	Cetuximab	33.8% [23.4–45.5]	5.0 mo [3.8–6.2]
Klute [45]	Vemurafenib	Cetuximab	30% [14–50]	15.7 wks [12.1–18.10
Tian [48]	Dabrafanib	Trametinib + sparatlizumab	24.3% [11.9–41.2]	4.3 mo [3.7–7.3]
*Central nervous system cancers*				
Wen [49]	Dabrafenib	Trametinib	HGG: 33% [20–49]LGG: 69% [39–91]	3.8 mo [1.8–9.2]
Hargrave [51]	Dabrafenib	Trametinib	56% [40–72]	9 mo [5.3–24]
*Lung cancer*				
Riely [55]	Encorafenib	Binimetinib	75% [62–85] treatment naïve / 46% [30–63] in previously treated patients	NE in treatment naive 9.3 mo in previously treated [6.2–NE]
Planchard [56]	Dabrafenib	Trametinib	68.4% [54.8–80.1]	10.2 mo [6.9–16.7]
Planchard [58]	Encorafenib	Binimetinib	66.7% [55–78.3]	11.1 mo [7.1–16.7]
Lin [59]	Vemurafenib	Cobimetinib	50%	7.9 mo [5.6–15.9]
Swalduz [60]	Dabrafenib	Trametinib	L1:73.8% [65.5–82.9] L2: 82.9% [71.4–94.4]	L1: 18.2 mo [7.7–21.3] L2: 10.4 mo [7.3–13.1]
Auliac [61]	Dabrafenib	Trametinib	NS	17.5 mo [7.1–23]
*Thyroid cancer*				
Subbiah [63]	Dabrafenib	Trametinib	69% [41–89]	NR
Tahara [64]	Encorafenib	Binimetinib	54.5% [32.2–75.6]	NR
*Biliary tract cancer*				
Subbiah [66]	Dabrafenib	Trametinib	51% [36–67]	NA
*Craniopharyngioma*				
Brastiasnos [67]	Vemurafenib	Cobimetinib	NS. DOPR 94% [70–100]	5.5 mo [3.7–9.6]

DNS = Dose not specified; mPFS = medial progression free survival; mDOR = median duration of response; ORR = overall response rate; HGG = high-grade glioma; LGG = Low-grade glioma; OS = overall survival; NR = Not reached; NS = not stated; DOPR = Durable objective partial response or better; mo = months; 5-FU = 5- flurouracil

In CNS tumours, responses to BRAF and MEK inhibitor combinations were more favourable in low-grade gliomas than high-grade gliomas. Wen et al. reported an ORR of 69% in low-grade gliomas compared to 33% in high-grade gliomas, with a median PFS of 3.8 months across groups [49].

Lung cancer studies showed higher response rates with dual inhibition regimens compared to monotherapy. Riely et al. observed an ORR of 75% in treatment naïve NSCLC patients receiving encorafenib and binimetinib [55]. Planchard et al. reported similar efficacy with dabrafenib and trametinib, with a median PFS of around 10 months [56]. Auliac et al. documented a median PFS of 17.5 months with the same regimen [61].

In thyroid cancers, Subbiah et al.’s study [63] demonstrated an ORR of 69% with dabrafenib and trametinib, while Tahara et al [64] reported an ORR of 54.5% with encorafenib and binimetinib. For biliary tract cancers, Subbiah et al. noted an ORR 51% [66]. Craniopharyngiomas were less commonly represented in trials but showed a durable objective partial response or better of 94% in a small cohort treated with vemurafenib and cobimetinib [67].

Basket trials assessing combination therapy, such as the ROAR study and NCI-MATCH subprotocols, further demonstrated the pan-cancer efficacy of BRAF inhibitors, particular for rare cancers. For instance, the ROAR trial reported durable responses in patients with biliary tract cancers, gliomas and anaplastic thyroid cancers, supporting the broader application of BRAF inhibition. ORRs were particularly notably in biliary tract cancers (51%), anaplastic thyroid cancers (69%) and gliomas (up to 56%) with several cohorts demonstrating prolonged disease control and PFS. Although the ROAR trial was a basket trial, many histotypes were reported in individual, single-tumour type studies, and have therefore been included in Table 6.

Hyman et al.’s basket trial demonstrated preliminary efficacy of vemurafenib in BRAF V600 mutated NSCLC, with partial responses observed in patients with anaplastic thyroid cancer (n = 2), cholangiocarcinoma (n = 1), ovarian cancer (n = 1), salivary duct cancer (n = 1) and sarcoma (n = 1) [36]. Patients with colorectal cancer had superior outcomes when treated with vemurafenib monotherapy compared with combination therapy with cetuximab. However, this cohort contained a high proportion of patients who had been previously treated with anti-EGFR antibodies (44%), possibly contributing to the unexpected outcome [36].

In the NCI-MATCH subprotocols (seen in Table 7), dabrafenib and trametinib were evaluated, albeit in a smaller cohort of patients. Durable responses were observed in lung, gastrointestinal and CNS malignancies.

**Table 7:** Basket trials efficacy (combination therapy)

Study	BRAFi	Combination therapy	Major tumour types	Outcome [95% CI]
Hyman [36]	Vemurafenib 960 mg BD	± cetuximab in a subpopulation of patients with CRC	Primary tumour, N (%) n = 122 NSCLC 20 (16) Primary brain tumour 13 (11) Cholangiocarcinoma 8 (7) ECD/Langerhan cell histiocytosis (LCH) 18 (15) Anaplastic thyroid cancer 7 (6) Multiple myeloma 5 (4) Colorectal cancer 37 (30)–27 of these patients received cetuximab in addition Others 14 (11) Others include breast and ovarian cancer, pancreatic cancer, CUP and anaplastic ependymoma	Stratified by tumour type: *NSCLC* ORR 42% [20–67] mPFS 7.3 mo [3.5–10.8] mOS NR *ECD/LCH* ORR 43% [18–71] 12 mo PFS 91% [51–99] *Colorectal cancer (vem monotherapy)* mPFS 4.5 mo [1–5.5] OS 9.3 mo [5.6–NR] *Colorectal cancer (vem* + *cetux)* mPFS 3.7 mo [1.8–5.1] OS 7.1 mo [4.4–NR]
Tan [68]	Vemurafenib 960 mg BD	Erlotinib	Primary tumour, N (%) n = 39 Colorectal cancer 32 (82) Breast cancer 1 (2) Malignant GIST 1 (2) Metastatic ovarian LGSC 1 (2) NSCLC 4 (10)	ORR 21% [NS] mOS NR mPFS 5.5mo [3–NR]
Tahara [69]	Dabrafenib 150 mg BD	Trametinib	Primary tumour, N (%), n = 57 Thyroid cancer 15 (26) Glial tumour of CNS 16 (28) Liver and intrahepatic duct 4 (7) Colorectal 4 (7) Non glial CNS tumour 3 (5) Pancreas 3 (5) Gallbladder and extrahepatic duct 2 (3) Other 10 (17) Others include melanoma, cancer of unknown primary, sarcoma, uterine cancer and ovarian cancer	ORR 28% [16.2–42.5] DCR 84% [70.9–92.8] PFS 6.5mo [4.2–7.2]
Salama [37]	Dabrafenib 150 mg BD	Trametinib	Primary tumour, N (%) n = 29 GI tract 11 (38) Lung adenocarcinoma 5 (17) Low grade serous ovarian carcinoma 5 (17) Mucinous-papillary serous adenocarcinoma of the peritoneum 1 (3) CNS 5 (15) Haematological 1 (3) Ameloblastoma of mandible 1 (3)	ORR: 37% [22.9–54.9] mDOR 25.1 mo [12.8–NA] mPFS 11.4 mo [7.2–16.3] mOS 28.6 mo

Note that values are rounded to the nearest 0.1 decimal point. ORR = overall response rate; OS = overall survival; DOR = duration of response; mPFS = median progression free survival; OD = once daily; BD = twice daily; DCR = disease control rate; PD = progressive disease; SD = stable disease; CR = complete response; PR = partial response; NR = not reached; NS = not stated

Overall, ORRs varied by tumour type and treatment regimen (ie monotherapy or combination therapy), with lung cancers, CNS tumours and thyroid cancers demonstrating the highest ORR. The median PFS varied widely, from 3.8 months in high-grade gliomas, to over 17 months in some lung cancer cohorts. Some studies reported durable responses and disease control even after therapy discontinuation and re-challenge, suggesting that consistent BRAF inhibition may play a role in treatment efficacy.

### Toxicities

The rates of ≥ grade 3 adverse events (AE) in studies assessing BRAF monotherapy varied greatly, ranging from 17% to 77%, as seen in Table 8. The most common AE reported included asthenia, rashes, pyrexia and deranged liver function tests. Most of the studies assessing toxicity data in monotherapy trials used standard dosing for adult patients (for example, 960 mg of vemurafenib) or weight-based dosing (particularly in paediatric patients). Therefore, we were unable to make meaningful conclusions on whether toxicities were dose dependent.

**Table 8:** Toxicities-monotherapy

Study	Dose	≥ Grade 3 adverse events
*Encorafenib*		
Tahara [64]	DNS	27%
*Vemurafenib*		
Kaley [50]	960 mg BD	17%
Subbiah [54]	960 mg BD	GR3-4: 77%
		GR5: 3%
Brose [65]	960 mg BD	65%
Mazieres [62]	960 mg BD	Asthenia (10 patients, 10%), cutaneous epidermoid carcinoma (8, 8%), dermatitis (6, 6%), and increased gamma glutamyl transpeptidase (GGT) levels (6, 6%)
Blay [70]	960 mg BD	50.5%
Subbiah [71]	DNS	73%
*Tovorafenib*		
Kilburn [30]	DV	42%
*Dabrafenib*		
Hargrave [52]	Weight based dosing	28%
Planchard [57]	150 mg BD	Cutaneous squamous cell carcinoma (12%), asthenia (5%), and basal cell carcinoma (5%)

DNS = Dose not specified; DV = Doses varied; NS = Not specified

Combination therapies were associated with higher toxicity rates in general (as seen in Table 9). Grade ≥ 3 adverse events were observed in 13%—75% of patients receiving encorafenib or vemurafenib in combination with MEK or EGFR inhibitors. Data on grade ≥ 3 AE in patients treated with dabrafenib and trametinib were reported in a less standardised manner, with overall rates less commonly reported in favour of the rates of individual AEs

**Table 9:** Toxicities–combination therapy

Study	Dose	Combination	≥ Grade 3 adverse events
Encorafenib			
Koptez [39]	DNS	Encorafenib, binimetinib, and cetuximab (triplet-therapy group); encorafenib and cetuximab (doublet-therapy group)	58% of patients in the triplet-therapy group, in 50% in the doublet-therapy group, and in 61% in the control group
Riely [55]	450 mg OD	Binimetinib	41%
Cutsem [41]	300 mg OD	Binimetinib ± cetuximab	Anaemia (11%), asymptomatic lipase increase (11%), diarrhea (10%), and nausea (8%)
Koptez [42]	DNS	Encorafenib (E) + cetuximab (C) ± chemotherapy (CT)	NS
Planchard [58]	450 mg OD	Binimetinib	The percentage of patients with at least one dose reduction was 34.4% for encorafenib and 32.8% for binimetinib (of which 90% for AE)
Fernandez Montes [44]	DNS	Cetuximab	13.5%
Gallois [47]	DNS	Cetuximab ± binimetinib	21%
Vemurafenib			
Hyman [36]	–	Cetuximab	Vem monotherapy: 73%. In vem + cetux, 74%
Brastiasnos [67]	960 mg BD	Cobimetinib	75%
Koptez [40]	960 mg OD	Chemotherapy	Grade 3 and 4 adverse events higher in the experimental arm than those in the control arm included: neutropenia (30% v 7%), anemia (13% v 0%), and nausea (19% v 2%). Eleven of 50 (22%) patients in the experimental arm discontinued treatment because of adverse events, compared with 4 of 50 (8%) in the control arm
Tan [68]	960 mg BD	Erlotinib	Diarrhea (26%) and acneiform rash (8%)
Ducreux [43]	950 mg BD	Cetuximab	60%
Lin [59]	960 mg BD	Cobimetinib	Ten patients (16%) discontinued treatment and 21 (33%) required dose adjustments due to adverse events
Klute [45]	DNS	Cobimetinib	43%
Chen [46]	DNS	Vem/irinoteca/cetux v beva + chemo	34.2% and 32.5% for the VIC regimen and bevacizumab plus chemotherapy respectively
Dabrafenib			
Wen [49]	150 mg BD	Trametinib	53%
Subbiah [66]	150 mg BD	Trametinib	GR3 or worse raised GGT: 12% serious adverse events (21%)
Subbiah [63]	150 mg BD	trametinib	Fatigue = 38%
			Nausea = 35%
			Pyrexia = 37%
Planchard [57]	150 mg BD	Trametinib	NS
Hargrave [51]	Weight based dosing	Trametinib	34.1%
Tahara [69]	150 mg BD	Trametinib	45.6%
Swalduz [60]	300 mg daily	Trametinib	Discontinuation for toxicity was reported in 10.3% of patients
Auliac [61]	150 mg BD	Trametinib	NS
Salama [37]	150 mg BD	Trametinib	The most common grade 3 AEs felt to be possibly related to treatment were fatigue, neutropenia, hyponatremia, and hypophosphatemia; there was 1 grade 4 sepsis
Tian [48]	DNS	Sparatlizumab (PDR001), and trametinib	Most common AE (grade 3 or greater) was increased lipase (8.1%), fever (5.4%), hyponatremia (5.4%) fatigue (2.7%), decreased neutrophil count (2.7%), anorexia (2.7%) and colitis (2.7%)

DNS = Dose not specified; DV = Doses varied; NS = Not specified

Up to 22% of patients in some trials had to discontinue therapy due to toxicity, especially those using triplet regimens or who were heavily pre-treated. Rates of AE with monotherapy and combination therapy were comparative to those described elsewhere in the literature [73–76].

## Discussion

###  Summary of findings

BRAF inhibition was first shown to be effective in BRAF-mutant melanoma, with seminal trials such as BRIM and the COMBI series demonstrating high ORR (~ 70%) and median PFS of approximately 11–15 months with combined BRAF and MEK inhibition [77–80].

While BRAF inhibition achieved comparable outcomes in certain non-melanomatous cancers, efficacy in other cancers was more limited. Moreover, the durability of responses in non-melanomatous cancers was generally shorter, with the notable exception of paediatric low-grade gliomas, where response rates exceeding 12 months had been reported [63]. Melanoma remains an outlier in its responsiveness not only to single agent BRAF inhibition, but also to a broad spectrum of targeted and immunotherapeutic approaches, reflecting distinctive biological and immunogenic characteristics of this tumour type [81–83].

This systematic review evaluated the efficacy and safety of BRAF inhibition, both as monotherapy and combination therapy, across non-melanomatous cancers harbouring BRAF mutations. A total of 36 studies, encompassing 3141 patients were included, with the most common tumour types being colorectal, lung, thyroid, CNS and biliary tract. Class I BRAF mutations were the most commonly evaluated, with very few trials including a significant portion of non-V600E mutated cancers.

There was also variation with some tumour types (e.g., gliomas and thyroid cancers) demonstrating higher response rate than others (e.g., colorectal cancer). For many of the rarer cancers included in this review such as neuroendocrine cancers, peritoneal cancers, CUP and sarcomas, the smaller sample sizes made it more difficult to derive meaningful and robust conclusions about their efficacy. Importantly, 91% (n = 2855) of all patients included in this review had either a lung cancer, CNS tumour, thyroid cancer or gastro-intestinal malignancy, leaving fewer than 10% of patients (n = 286) making up all other tumour types including rarer cancers or CUP. Toxicity profiles across the reviewed studies were largely consistent with the known adverse event spectrum of BRAF ± MEK inhibition [80, 84, 85].

Overall, the findings of this review suggest that BRAF-targeted therapies have clinically meaningful activity across multiple non-melanomatous, BRAF-mutant tumours, but that this activity is not uniform across histologies. Differences in efficacy between tumour types, together with the variable benefit of monotherapy versus combination therapy, support a model of broad but histology-modulated efficacy rather than a truly tumour-agnostic effect.

### Limitations

Interpretation of the available evidence requires explicit consideration of heterogeneity across the included studies. The observed variability in efficacy is likely multifactorial, reflecting differences in tumour lineage, treatment strategy, mutation subtype and study design. These sources of heterogeneity reflect the predominance of early-phase studies and uneven representation of tumour types. Accordingly, these findings are best interpreted as evidence of differential activity across biological and clinical contexts rather than as a unified estimate of effect, as discussed in Table 10 below.

**Table 10:** Key sources of heterogeneity and their implications for interpretation

Source of heterogeneity	Evidence from this review	Implication
Tumour lineage	Most patients had colorectal, lung, thyroid or CNS tumours; rarer cancers and CUP were represented by small subgroups	Activity appears broad but histology-modulated, not uniform across tumour types
Treatment regimen	Studies included monotherapy, doublet and triplet regimens. Combination therapy generally showed higher ORR and longer PFS than monotherapy	Outcomes should be interpreted by regimen; pooling monotherapy and combination therapy may obscure meaningful differences
BRAF mutation subtype	Most studies were dominated by V600E disease; non-V600 alterations were infrequently represented	Conclusions are most applicable to V600E-mutant tumours and should be extrapolated cautiously to non-V600 disease
Study design	The evidence base was predominantly early-phase, non-randomised basket trials, with limited randomised data	Efficacy signals are encouraging but vulnerable to bias, imprecision and overestimation
Rare tumour representation	Several rare histologies showed promising signals, but patient numbers were very small	Findings in rare cancers should be considered hypothesis-generating rather than definitive

Most comprised phase II trials, retrospective cohorts, or basket designs, frequently with limited sample sizes in rare tumour types. The paucity of RCTs reflects the ethical and logistic challenges inherent to evaluating tumour-agnostic therapies in biomarker-defined subgroups. The absence of matched control arms in most studies further constrained comparative interpretation. Together, these factors increase the risk that the reported efficacy may be overestimated, particularly when rare histologies have wide confidence intervals.

Interpretation was constrained by inconsistent reporting of efficacy endpoints across studies. Outcomes such as ORR, PFS, OS and DOR were not uniformly defined or reported across studies, limited direct comparability and precluding more standardised synthesis. Generalisability was limited by the review methodology. Literature screening, selection and extraction were conducted predominantly by a single reviewer., which may have introduced risk of extraction bias. Generalisability was further constrained by the distribution of tumour types. Although this review addresses tumour-agnostic applications of BRAF inhibition, the evidence base was dominated by more common malignancies, whereas rarer cancers and CUP were represented by a small number of patients.

### Contextualising tumour-agnostic therapies: broad activity, histology-modulated efficacy

Although tumour-agnostic therapies are conceptually defined by activity across cancers sharing a molecular alteration, the findings of this review suggest that BRAF-directed therapy is better understood as targeting a context-dependent signalling dependency rather than a uniformly histology-independent vulnerability. The clearest example is colorectal cancer, where limited monotherapy activity and improved outcomes with EGFR- and MAPK-directed combinations are consistent with adaptive feedback through parallel receptor tyrosine kinase signalling.

By contrast, the more reproducible activity observed in selected lung, thyroid and CNS tumours suggests that, in at least some histologies, oncogenic signalling remains more tightly coupled to BRAF-MAPK pathway output. Even within CNS tumours, however, the higher activity seen in low-grade compared with high-grade glioma, together with rapid progression off therapy and response on rechallenge in paediatric low-grade glioma, suggests that therapeutic effect may reflect sustained pathway suppression rather than uniform tumour cell eradication.

Similarly, higher response rates in treatment-naïve than previously treated NSCLC imply that pathway dependence may be modified by treatment history and acquired adaptive circuitry. Taken together, these observations suggest that BRAF mutation status functions less as a universally portable tumour-agnostic biomarker and more as a conditional biomarker of pathway dependence, the predictive value of which is modified by lineage, co-alteration landscape, and the availability of rational combination strategies.

### Future directions

The findings of this review highlights both the potential and limitations of tumour-agnostic applications of BRAF inhibition. Although BRAF mutations are evident across a variety of rare and common tumours, their clinical efficacy may be impacted by individual and varied TMEs, co-occurring mutations and resistance mechanisms. This biological heterogeneity challenges the concept of a truly uniform, histology-independent therapeutic effect.

A key finding from this review is the recognition that lack of efficacy of a single targeted agent should not preclude investigation of combination strategies in specific histologies. In addition, although this review highlights the influence of histological context on treatment efficacy, several rare tumour types, such as pleomorphic xanthoastrocytoma, sarcomas and ovarian carcinomas demonstrated encouraging responses to BRAF inhibition in early phase or small cohort studies. This suggests that a potentially broader scope of benefit may exist that may not be fully captured due to under-representation in clinical trials. Further given rare cancers collectively represent a significant proportion of the cancer burden, a more appropriate strategy may be to design dedicated tumour-agnostic trials, such as the ROAR trial, which specifically enrol patients with rare cancers and CUP.

Future work should therefore focus less on whether BRAF inhibition is strictly tumour-agnostic, and more on defining the biological settings in which BRAF-directed therapy is most effective as monotherapy, and those in which histology-specific combination strategies are required.

## Conclusion

Currently, clinical decision-making in this space remains largely guided by early-phase, non-randomised trials with small cohorts and limited comparator data. Within these constraints, the available evidence suggests that BRAF-targeted therapy has meaningful activity across non-melanomatous tumours, particularly when used in rational combination regimens, but the magnitude of benefit varies by tumour type and biological context.

Rather than supporting a uniformly tumour-agnostic effect, the literature is more consistent with a model of broad but histology-modulated efficacy. Further studies should prioritise more structured, tumour-specific and regimen-specific evaluation, with incorporation of translational endpoints such as resistance mechanisms and co-mutation profiling, to better define which patients are most likely to benefit.

## Abbreviations

AE: Adverse events
ALK: Anaplastic lymphoma kinase
BRAF: V Raf Murine Sarcoma Viral Oncogene Homolog
BRAFi: BRAF inhibition
CI: Confidence interval
CNS: Central nervous system
CUP: Cancer of unknown primary
DCR: Disease control rate
DNS: Dose not specified
DOR: Duration of response
DV: Doses vary
ECD: Erdheim–Chester disease
EGFR: Epidermal growth factor receptor
EMA: European Medical Association
ERK: Extracellular signal-regulated kinase
ESMO: European Society of Medical Oncology
ETAC-S: ESMO Tumour Agnostic Classifier and Screener
FDA: US Food and Drug Administration
HER2: Human epidermal growth factor receptor 2
HLA: Human leukocyte antigen
ICER: Incremental cost effectiveness ratio
ICI: Immune checkpoint inhibitors
LHC: Langerhans histiocytosis
MAPK: Mitogen-activaed protein kinase
MEK: Mitogen-activated protein kinase kinase
MeSH: Medical subject heading terms
MGTOs: Molecularly guided treatment options
MMR: Mismatch repair
MSI: Microsatellite instability
NGS: Next generation sequencing
NICE: National Institute of Health and Care Excellence
NS: Not specified
NSCLC: Non small cell lung cancer
NTRK: Neutrophic tyrosine receptor kinase
OR: Objective response
ORR: Overall response rate
OS: Overall survival
PBS: Pharmaceutical Benefits Scheme
PCR: Polymerase chain reaction
PFS: Progression free survival
PRISIMA: Preferred reporting items for systematic reviews and meta-analysis
QALY: Quality adjusted life years
QOL: Quality of life
RAS: Rat sarcoma
ROS: Receptor tyrosine kinase-like orphan receptor
TGA: Therapeutic Goods Administration
TMB: Tumour mutational burden
TME: Tumour microenvironment
